# Nanoparticle albumin-bound paclitaxel -induced severe interstitial lung disease in a gastric cancer patient: a case report

**DOI:** 10.3389/fonc.2026.1792694

**Published:** 2026-02-26

**Authors:** Baosheng Liu, Kaihui Wei, Jianru Dong, Xiuli Zhou

**Affiliations:** 1 Department of Clinical Pharmacy, Weifang Hospital of Traditional Chinese Medicine, Weifang, China; 2 Department of Clinical Pharmacy, Weifang People’s Hospital, Weifang, China

**Keywords:** adverse drug reaction, case report, gastric adenocarcinoma, interstitial lung disease, nab-PTX

## Abstract

**Background:**

Nanoparticle albumin-bound paclitaxel (nab-PTX) is a novel formulation that combines paclitaxel with human serum albumin via nanotechnology. Without the need for solubilizers, it allows for higher safe doses, shorter infusion time, and no premedication for hypersensitivity prevention. nab-PTX has been widely used in the treatment of various solid tumors. Its common adverse reactions include fatigue, alopecia, myelosuppression, etc., while pulmonary toxicity is extremely rare. To date, there are no reports of severe drug-induced interstitial lung disease (DILD) caused by nab-PTX in gastric cancer patients.

**Case presentation:**

We report a case of a 77-year-old male patient with gastric adenocarcinoma. On the 15th day after receiving second-line nab-PTX monotherapy, the patient developed chest tightness, dyspnea, high fever, and severe respiratory distress. High-resolution computed tomography (HRCT) of the chest showed diffuse exudative changes in both lungs, involving more than 90% of the lung fields. After excluding other causes such as pulmonary infection and tumor progression, the patient was diagnosed with severe nab-PTX-related DILD. nab-PTX was discontinued immediately, and the patient was treated with intravenous methylprednisolone sodium succinate. Subsequently, the pulmonary inflammation was gradually absorbed, and the clinical symptoms were significantly improved.

**Conclusion:**

nab-PTX-induced severe interstitial pneumonia in gastric cancer patients is a rare and life-threatening adverse event. Clinicians should closely monitor respiratory symptoms and signs of patients receiving nab-PTX, achieve early identification and timely intervention, so as to reduce the risk of adverse outcomes and improve patient prognosis.

## 1 Introduction

Gastric cancer is the fifth leading cause of malignancy-related death worldwide, with more than 1 million new cases diagnosed annually. Current standard first-line treatment for advanced gastric cancer includes platinum-based drugs combined with fluoropyrimidines or immune checkpoint inhibitors, while second-line treatment mainly adopts regimens of irinotecan, ramucirumab plus paclitaxel, and single-agent paclitaxel (1). nab-PTX is less likely to induce hypersensitivity reactions due to the absence of polyoxyethylene castor oil, and has been widely used in the treatment of various malignant solid tumors. Its common adverse reactions include fatigue, alopecia, myelosuppression, nausea and vomiting, and peripheral neuropathy. However, reports on its pulmonary toxicity remain extremely scarce; the limited existing cases have mostly focused on lung cancer patients, and relevant documentation in patients with other tumor types is particularly rare (2). This report presents an extremely rare case of severe DILD occurring in a gastric adenocarcinoma patient 15 days after nab-PTX treatment, aiming to provide a vital clinical reference for the accurate monitoring of patients’ symptoms and signs, prompt identification and diagnosis of DILD, and early initiation of targeted interventions to optimize patient prognosis.

## 2 Case report

A 77-year-old male patient was admitted to hospital on September 30, 2023, due to vomiting complicated with hematemesis, and was diagnosed with gastric adenocarcinoma by gastroscopic pathological examination. The first cycle of Xelox (oxaliplatin plus capecitabine tablets) chemotherapy was initiated on October 7, 2023. On November 2, 2023, the second cycle of Xelox combined with sintilimab injection for immunotherapy was administered, followed by sequential Xelox plus sintilimab immunotherapy every 21 days thereafter. The sixth cycle of this regimen was delivered on January 30, 2024, and the treatment was completed to enter the observation phase. A follow-up chest HRCT scan on March 1, 2024, revealed no obvious abnormalities in the lungs (**Figure 1A**). The patient was readmitted for examination on April 29, 2024, and tumor progression was identified; consequently, second-line monotherapy with nab-PTX was planned. Physical examination on admission showed symmetrical thoracic cage, clear breath sounds over both lungs, no audible rales or rhonchi, and absence of pleural friction rub. Chest HRCT scan demonstrated no obvious pulmonary abnormalities (**Figure 1B**).

**BAFigure 1:**
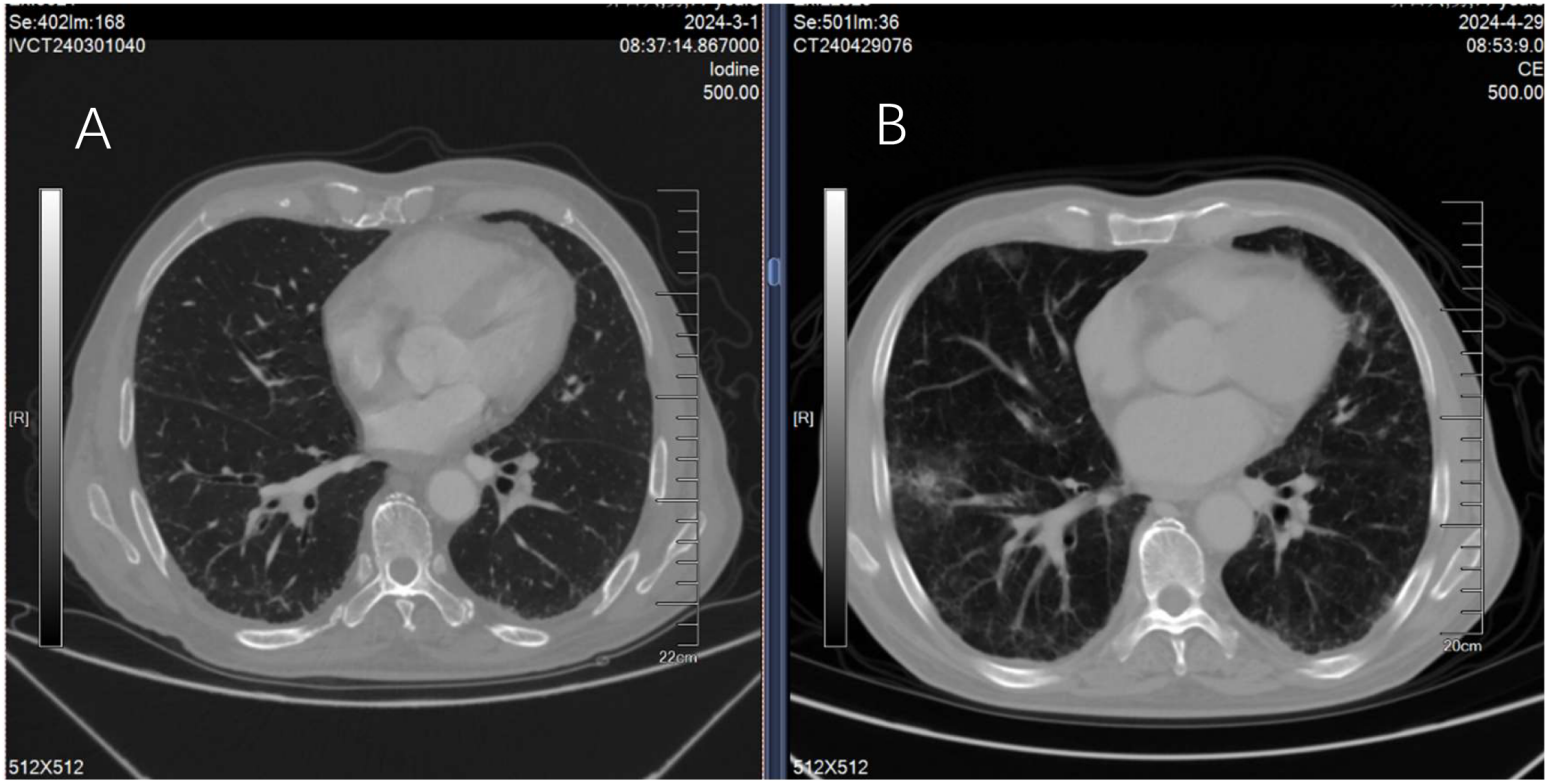
Chest HRCT examinations performed 2 months before and 1 day before nab-PTX treatment revealed no significant abnormal findings in both lungs **(A, B)**. nab-PTX monotherapy was initiated on April 30, 2024, at a dose of 100 mg via intravenous infusion (administered on day 1 and day 8, repeated every 21 days). The patient exhibited good tolerance during chemotherapy, with no obvious adverse reactions other than significant asthenia. During the chemotherapy interval on May 4, physical examination revealed clear breath sounds over both lungs, no audible rales or rhonchi, and absence of pleural friction rub. The second cycle of nab-PTX (100 mg, intravenous infusion) was administered on May 7. The patient maintained independent ambulation during treatment, with no cough or expectoration; auxiliary examinations showed normal inflammatory markers including white blood cells and neutrophils.

On May 13, the patient developed intermittent chest tightness and dyspnea, being only capable of mild activity, complicated with high fever (maximum body temperature of 39.3 °C) accompanied by chills, intermittent cough with scanty sputum. Indomethacin suppository (0.1 g) was then administered per anum for antipyretic therapy. Auxiliary examinations demonstrated elevated inflammatory markers above the normal range (hypersensitive C-reactive protein: 79.72 mg/L, procalcitonin: 0.186 ng/mL); the oxygen saturation was 88% in room air, and nasal cannula oxygen inhalation was initiated at a flow rate of 5 L/min. Urgent investigations, including sputum culture, fungal sputum culture, galactomannan (GM) assay, influenza virus nucleic acid detection via pharyngeal swab, pharyngeal swab culture, Epstein-Barr virus (EBV) DNA testing, cytomegalovirus (CMV) DNA testing, anti-tuberculosis antibody assay, acid-fast bacillus (AFB) smear and culture of sputum, and a panel of ten infectious disease screening tests, yielded no significant abnormal findings.

**Figure 2:**
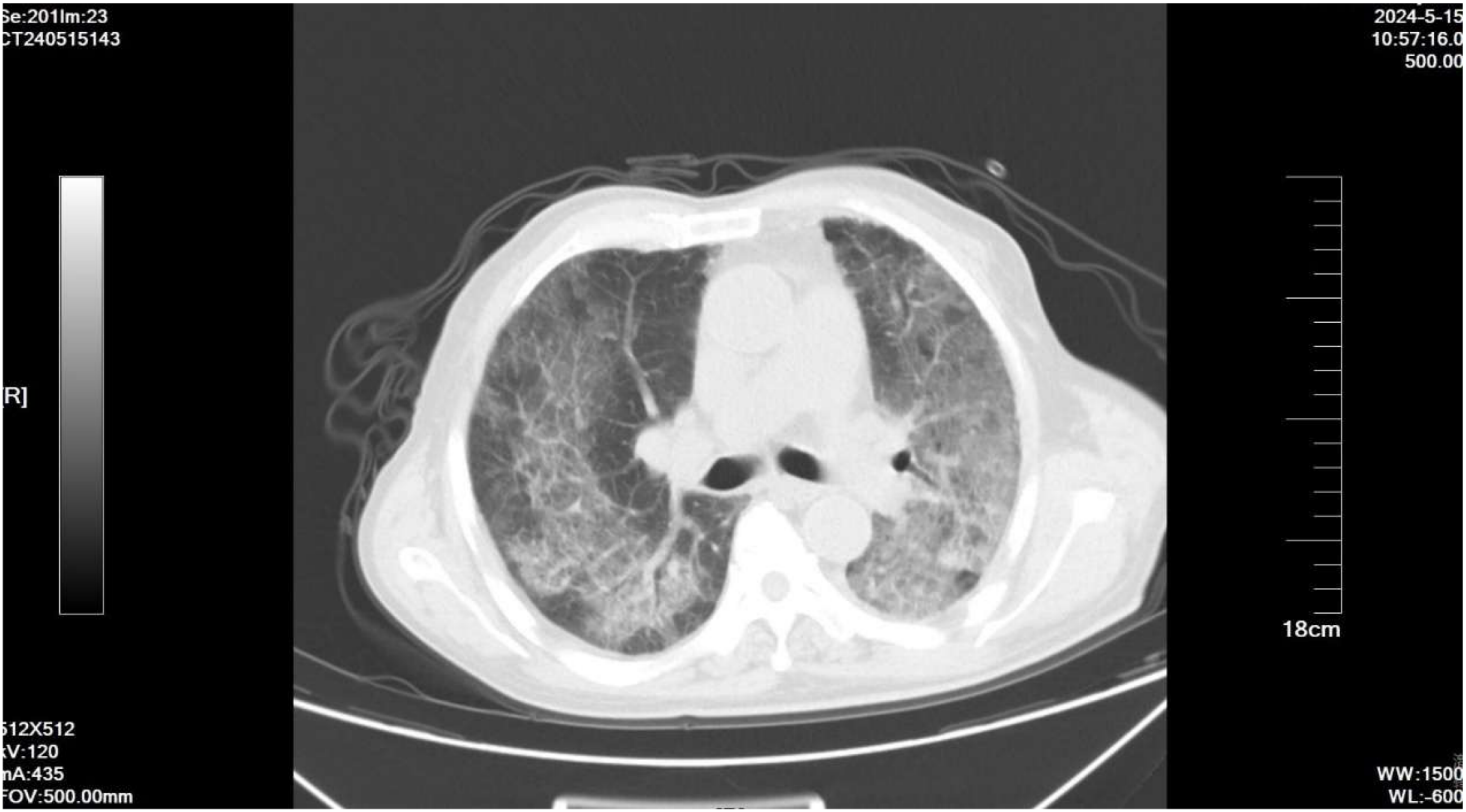
HRCT of the chest obtained 15 days after the first dose of nab-PTX revealed diffuse exudative changes in the bilateral lung parenchyma, accompanied by extensive ground-glass opacities and consolidation, involving more than 90% of the bilateral lung fields.

**Figure 3:**
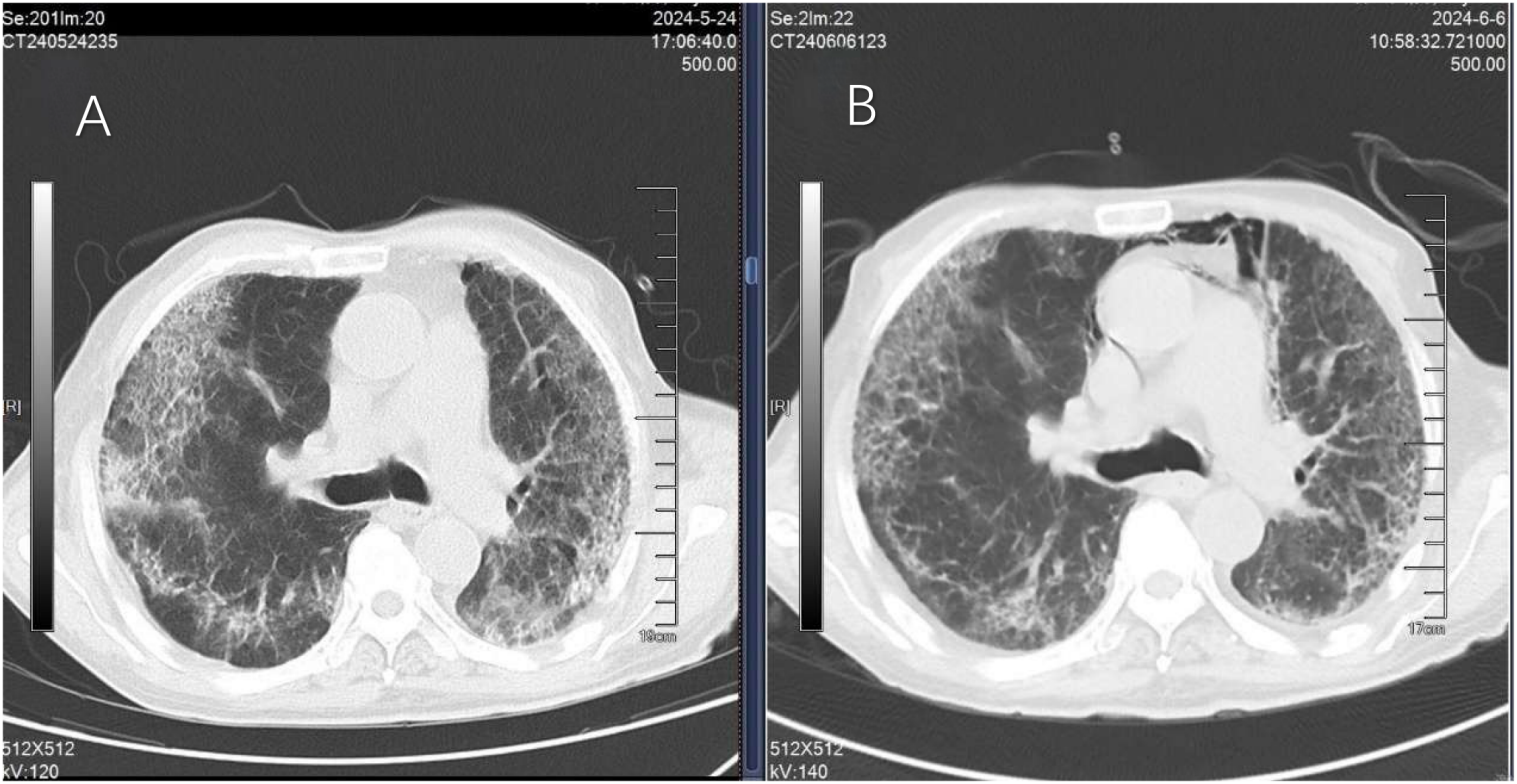
Chest HRCT obtained 9 days after glucocorticoid therapy revealed partial absorption and resolution of extensive ground-glass opacities and consolidation in both lungs, with reduced pulmonary fibrosis **(A)**. Chest HRCT examination 22 days after glucocorticoid therapy revealed a significant reduction in the extent of bilateral lung lesions and a marked improvement in lesion severity **(B)**.

**Figure 4:**
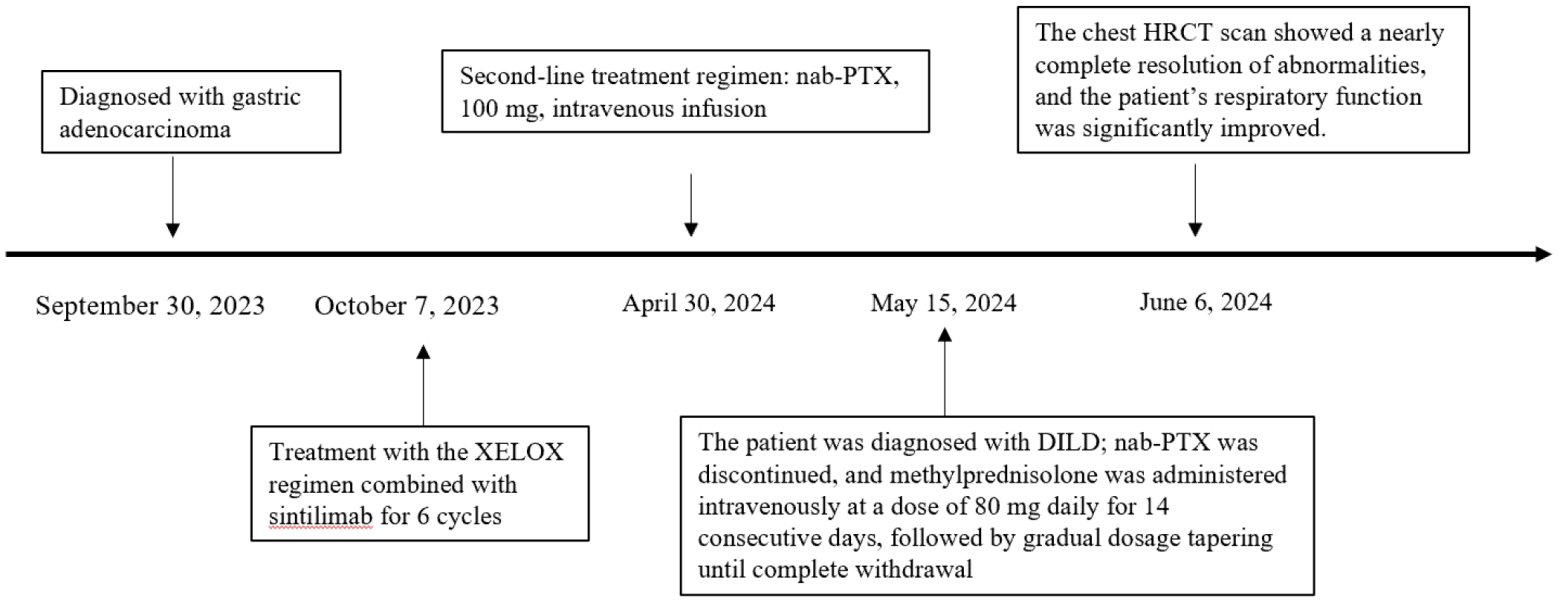
Timeline depicting the patient’s clinical course, including diagnosis, first-line and second-line anti-tumor therapy, development of DILD, glucocorticoid administration, and effective control of adverse reactions.

On May 15, the patient presented with severe dyspnea accompanied by bilateral pulmonary moist rales. Chest HRCT revealed diffuse parenchymal exudative changes in both lungs, involving more than 90% of the lung fields (**Figure 2**). The patient presented with severe dyspnea and orthopnea, which was exacerbated by minimal activity; the patient declined invasive examinations including bronchoscopy with bronchoalveolar lavage and lung biopsy. nab-PTX was discontinued on May 15, and intravenous infusion of methylprednisolone sodium succinate injection was initiated at a dose of 80 mg daily for 14 consecutive days, followed by a gradual tapering of the dosage until withdrawal (30 mg daily for 5 days, then 25 mg daily for 5 days). A follow-up chest HRCT on May 24 showed a certain degree of improvement in bilateral pulmonary inflammation compared with the prior scan (**Figure 3A**). Repeat chest HRCT on June 6 demonstrated significant absorption of bilateral pulmonary inflammation (**Figure 3B**); the patient’s dyspnea and chest tightness were substantially relieved, with recovery of independent mild activity and absence of cough or expectoration. At the follow-up visit on June 20, physical examination revealed clear breath sounds over both lungs with no audible rhonchi or rales, and the patient had no dyspnea following mild activity. Given the progression of the patient’s primary disease and intolerance to the second-line treatment regimen, third-line targeted therapy was initiated with oral apatinib mesylate tablets. On September 13, the patient’s primary disease progressed further, and apatinib mesylate was discontinued. Following full communication with the patient and their family, subsequent treatment was palliative care-oriented, aiming to improve the patient’s quality of life. **Figure 4** illustrates the treatment timeline of the patient.

## 3 Discussion

This case focuses on a gastric cancer patient who developed severe DILD following nab-PTX administration, an adverse event that is exceedingly rare and life-threatening in gastric cancer patients and carries important clinical warning implications. A comprehensive search of the PubMed, Web of Science, and Embase databases was conducted using the terms “Nanoparticle Albumin-Bound Paclitaxel,” “case report,” “adverse,” “induced,” and “related.” Analysis of the retrieved cases indicated that nab-PTX-related ADRs predominantly affect the hematological and lymphatic systems (e.g., neutropenia), nervous system (e.g., peripheral neuropathy), musculoskeletal system (e.g., arthralgia/myalgia), and gastrointestinal system (e.g., nausea, vomiting, and constipation) (2, 3). There are also a few relevant research reports on nab-PTX-induced DILD in lung cancer patients, and such adverse events have been documented in large-scale clinical trials (4, 5). For example, a clinical study in Europe and the United States reported that respiratory system-related adverse reactions occurred in patients receiving nab-PTX in the European and American populations: 12% experienced dyspnea, 7% had cough, and pneumothorax was relatively rare (< 1%). In the Chinese population, only 2% developed dyspnea and cough (6). In previous safety studies on nab-PTX, reports of DILD are relatively rare and mainly concentrated in lung cancer patients and a small number of breast cancer patients, with no clinical reports of nab-PTX-induced severe DILD in gastric cancer patients to date. However, DILD is a potentially fatal complication in cancer treatment and still requires attention in clinical practice.

DILD induced by taxanes is clinically rare and unpredictable. Studies have shown that the incidence of paclitaxel-associated DILD ranges from 3% to 12%, with that of severe cases of grade 3 or higher at 0.73% (7); the incidence of DILD caused by nab-PTX is 1.5% to 4.5%, and that associated with docetaxel is 4.6% (8). A retrospective study by Kashiwada et al. (9) confirmed that pre-existing pulmonary comorbidities (e.g., a history of chronic obstructive pulmonary disease or interstitial changes on computed tomography) are risk factors for nab-PTX-related DILD: among 110 patients with advanced lung cancer treated with nab-PTX, 9 (8.2%) developed DILD, with the incidence reaching 19.0% (8/42) in patients with a prior history of interstitial pneumonia and only 1.5% (1/68) in those without such a history. In addition, the incidence of pulmonary toxicity in patients with malignant tumors is closely associated with tumor type: lung cancer patients have poor pulmonary reserve, and even mild deterioration of pulmonary function may lead to prominent clinical manifestations; in contrast, patients with other tumor types generally have well-preserved pulmonary reserve (except for those with comorbidities), and interstitial pneumonia may present with atypical features, making it prone to misdiagnosis.DILD refers to a group of diffuse pulmonary disorders affecting the pulmonary interstitium, alveolar spaces, or bronchioles, which is typically characterized by severe pulmonary function impairment and hypoxemia. The radiological criteria for DILD include the presence of reticular opacities, irregular linear opacities, and honeycombing in the peripheral subpleural regions of the lower lung lobes. The disease course of DILD induced by different types of drugs varies considerably, which may manifest shortly after drug administration (e.g., within several days to weeks) or develop insidiously (e.g., months after treatment initiation) (10). DILD lacks specific clinical manifestations. Mild cases may be asymptomatic and only detected incidentally on radiological examinations. As the disease progresses, patients may present with dry cough and progressively worsening exertional dyspnea; some patients may develop systemic symptoms such as asthenia, fever, and rash (11). DILD also has no specific physical signs, with possible findings including tachypnea and cyanosis of the lips and oral mucosa. Lung auscultation is usually unremarkable, while a small number of patients may have audible moist rales or Velcro rales. For patients with pre-existing pulmonary underlying diseases, the development of exacerbated respiratory symptoms and/or signs during antineoplastic drug treatment warrants a thorough evaluation to rule out DILD (12).To date, there are no specific radiological, serological, or pathological tests for the diagnosis of DILD. Nevertheless, radiological imaging plays a pivotal role in the diagnosis of DILD, and chest HRCT, in particular, is critical for assessing pulmonary abnormal findings, lesion extent, and serial follow-up monitoring. Prior to the administration of second-line antineoplastic agents, the patient had unremarkable findings on bilateral lung auscultation and chest HRCT. On day 13 after the first administration of nab-PTX, the patient developed chest tightness, fatigue, limited activity, a high fever of up to 39.3 °C with chills, intermittent cough, and scanty sputum; on day 15, severe dyspnea and bilateral pulmonary rales appeared, and chest HRCT revealed extensive ground-glass opacities and airspace consolidation in both lungs, which were consistent with the clinical and radiological features of DILD. During first-line antineoplastic therapy, the patient received a total of five cycles of sintilimab-based immunotherapy. Although sintilimab carries a potential risk of inducing immune-related pneumonitis, physical examinations throughout the entire treatment course revealed clear breath sounds over both lungs, with no audible rhonchi, rales or pleural friction rubs; additionally, chest HRCT scans performed at 1 and 3 months after treatment completion showed no significant abnormalities, indicating a low temporal association between sintilimab and the current pulmonary lesions. Furthermore, the radiological features of sintilimab-associated immune-related pneumonitis (13) are predominantly characterized by multifocal patchy consolidations with a peribronchovascular and/or peripheral distribution, and a reversed halo sign may be present in some cases with mild degree of fibrosis—findings that are somewhat distinct from those observed in the present patient. Based on the above analysis of temporal association and radiological characteristics, it is inferred that the current pulmonary lesions are unlikely to be associated with sintilimab. In accordance with the diagnostic criteria for DILD defined by the Fleischner Society: radiological examinations reveal newly developed pulmonary lesions; a positive temporal association is identified between pulmonary lesions and drug exposure; alternative etiologies for pulmonary lesions are excluded (14). After ruling out pulmonary infection, tumor progression, radiation pneumonitis, pulmonary hemorrhage, pulmonary edema, and pulmonary disorders secondary to dysfunction of other organs, a causality assessment was performed using the Naranjo Causality Assessment Scale, with the patient in this case scoring 8 points. Following discussion by the multidisciplinary team (MDT), the DILD was ultimately determined to be probably attributable to nab-PTX. According to the pneumonia grading criteria of the National Cancer Institute Common Terminology Criteria for Adverse Events (NCI-CTCAE), the patient presented with impaired self-care ability and required oxygen therapy, which was indicative of severe symptoms and thus classified as Grade 3 (G3) (CTCAE G3) (15).

The pathogenesis of DILD caused by antineoplastic agents has not been fully elucidated to date. Studies have postulated that its pathological process may be closely associated with two major pathways: direct cytotoxic injury induced by the drug and immune-mediated tissue damage (16, 17). On the one hand, cytotoxic antineoplastic drugs can directly attack type I alveolar epithelial cells, capillary endothelial cells, and airway epithelial cells in lung tissue, impairing the normal structure and physiological function of the lung parenchyma. On the other hand, some drugs may act as haptens or mimic endogenous host antigens to activate immune cells, thereby triggering an immune-inflammatory response. Notably, these two pathogenic mechanisms do not exist independently; their effects are jointly regulated by multiple host and environmental factors, including age, underlying pulmonary diseases, drug metabolic characteristics, and genetic susceptibility of immune-related genes, which ultimately synergistically contribute to the onset and progression of DILD.

To date, the treatment of DILD has not been standardized, but typically involves discontinuing the suspected causative agent and suppressing the inflammatory response to control pulmonary interstitial fibrosis. For patients with CTCAE Grade 2–4 pulmonary toxicity, it is recommended to discontinue the suspected drug and initiate oxygen therapy and corticosteroid treatment. Previous studies have reported that glucocorticoids can alleviate symptoms and promote the repair of lung injury in moderate-to-severe DILD and acute exacerbation of DILD (18). However, data from large-sample clinical studies supporting the dosage and duration of glucocorticoid administration in DILD treatment are lacking, and no relevant clinical trials have clearly confirmed the definitive efficacy of corticosteroid therapy in DILD patients. The glucocorticoid treatment strategy for the patient in this case was primarily based on the *Expert Consensus on the Diagnosis and Management of Antineoplastic Drug-Related Interstitial Lung Disease* (19): high-dose glucocorticoid pulse therapy was administered in the initial treatment phase, and the dosage was gradually tapered in a stepwise manner until discontinuation after the clinical symptoms were effectively controlled. Relevant literature has reported (20) that in patients with a favorable response to glucocorticoid therapy, dyspnea typically resolves markedly within 1–2 weeks, with chest HRCT demonstrating significant improvement in pulmonary lesions; in contrast, non-responders experience progressive exacerbation of dyspnea. Following two weeks of high-dose glucocorticoid therapy, the patient’s symptoms were rapidly controlled, and the dosage was subsequently tapered stepwise over 10 days until complete discontinuation, with sustained and effective improvement in pulmonary symptoms. This treatment regimen may serve as a clinical reference for the management of DILD in subsequent cases.

In the practical clinical application of antineoplastic agents, individualized treatment plans can be formulated with reference to the relevant principles of DILD grading management, considering the patient’s underlying pulmonary diseases, comorbidity types, severity of adverse reactions, and tolerance to corticosteroids, thereby reducing the risk of potential complications. For complex cases or those with therapeutic difficulties, consultation with pulmonologists and endocrinologists may be requested, or a MDT discussion may be organized.

## 4 Conclusion

Herein, we report a rare case of nab-PTX-induced DILD in a gastric cancer patient. The pathogenesis of this condition remains unclear to date, and there are no specific clinical symptoms, signs, radiological findings, serological markers, or pathological features, making it prone to misdiagnosis and missed diagnosis. In clinical practice, healthcare providers should maintain a high degree of vigilance for DILD, identify high-risk populations early, and implement early prevention, early detection, early diagnosis, and early treatment. This approach will help reduce the incidence and mortality of DILD, improve patient prognosis, prolong survival, and further enhance the quality of life of cancer patients.

## Data Availability

The original contributions presented in the study are included in the article/supplementary material. Further inquiries can be directed to the corresponding author.
